# Absolute structure determination of compounds with axial and planar chirality using the crystalline sponge method†
†Electronic supplementary information (ESI) available: Details of sample preparation and crystallographic analysis. CCDC 1051799, 1051800, 1051618, 1051619, 1043948 and 1043949. For ESI and crystallographic data in CIF or other electronic format see DOI: 10.1039/c5sc01681a


**DOI:** 10.1039/c5sc01681a

**Published:** 2015-05-12

**Authors:** Shota Yoshioka, Yasuhide Inokuma, Manabu Hoshino, Takashi Sato, Makoto Fujita

**Affiliations:** a Department of Applied Chemistry , School of Engineering , The University of Tokyo , 7-3-1 Hongo, Bunkyo-ku , Tokyo 113-8656 , Japan . Email: mfujita@appchem.t.u-tokyo.ac.jp; b Life Science Square , Lab Solutions , Agilent Technologies Japan, Ltd. , Osaka University , TechnoAlliance Complex 3F, 2-8 Yamadaoka , Suita , Osaka 565-0871 , Japan

## Abstract

The absolute stereochemistry of compounds with axial and planar chirality is successfully determined by the crystalline sponge method without crystallization or derivatization of the compounds. This method is applied to absolute structure determination in the asymmetric synthesis of unique compounds with axial and planar chirality.

## Abstract 2

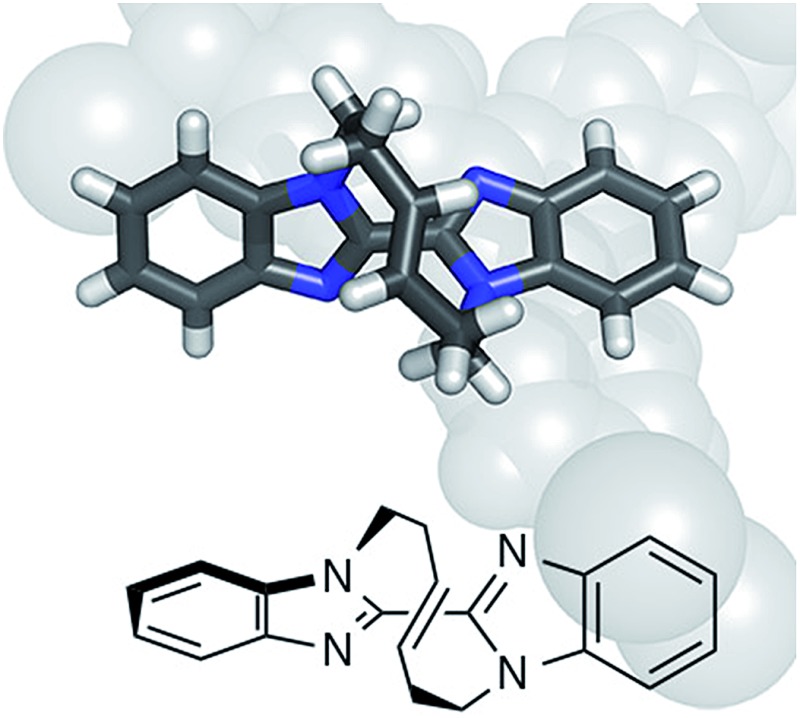
The absolute structure determination of compounds with axial and planar chirality obtained by recently developed asymmetric syntheses was achieved using the crystalline sponge method without using any reference compounds or synthetic modifications.

## Introduction

Chiral molecules with axial or planar chirality are of special interest to synthetic chemists because of both their unique chirality without stereogenic centers and their practical use as chiral auxiliaries or chiral ligands for catalytic asymmetric syntheses.^1^ There are therefore a number of reports on the asymmetric synthesis of these chiral molecules. Unlike common chiral molecules with stereogenic carbons, molecules with axial or planar chirality are not easily synthesized from or derivatized to known chiral compounds, and thus the determination of their absolute configuration is often troublesome. The Bijvoet method, which uses anomalous scattering in single crystal X-ray analysis, is one of the most common methods of determining the absolute chirality of chiral compounds,^2^ but the incorporation of heavy atoms is necessary and the compounds must then be crystallized.

**Fig. 1:**
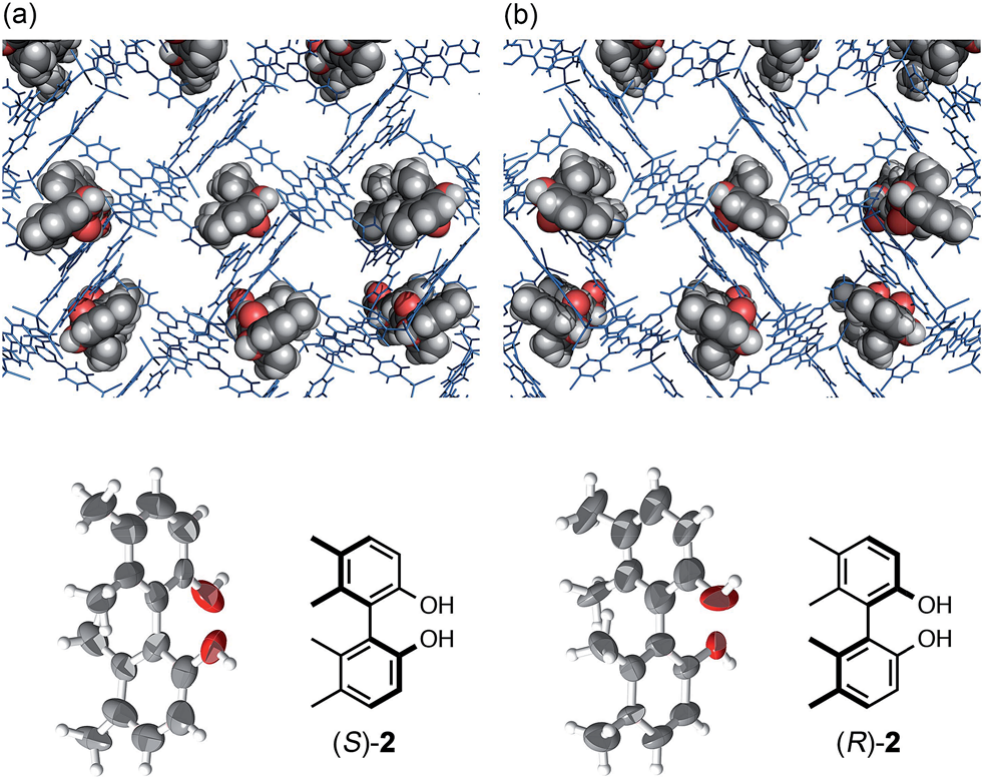
Crystal structures of (a) axially chiral (*S*)-**2** and (b) its enantiomer (*R*)-**2** determined by the crystalline sponge method (top: network structures with guest **2**, bottom: ORTEP drawings of guest **2** at 50% probability).

**Scheme 1:**
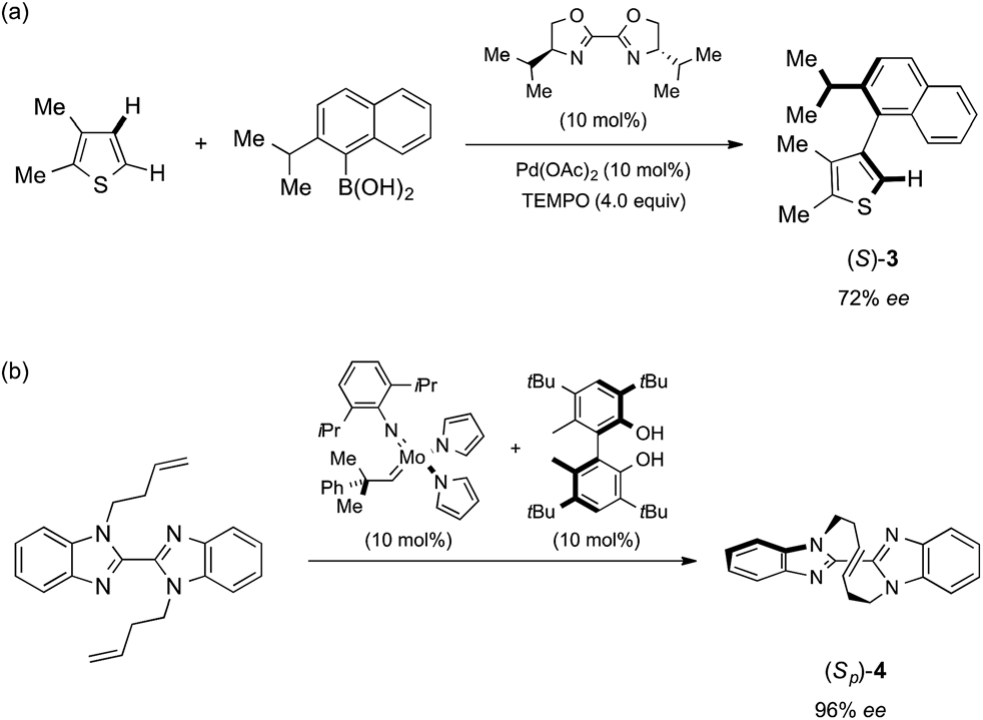
Reported asymmetric synthesis of (*S*)-**3** and (*S*_p_)-**4**. (a) Asymmetric cross-coupling *via* C–H activation developed by Yamaguchi and Itami.^8^ (b) Asymmetric olefin metathesis developed by Mori and Ogasawara.^9^

The crystalline sponge method is a recently developed technique for single crystal diffraction (SCD) studies that does not require crystallization of the samples.^3^ By soaking guests into porous coordination networks (crystalline sponges), the guests are oriented for SCD study.^4^ For the practical use of this method, a detailed protocol^5^ and experimental guidelines^6^ have been reported. When the sponge method is combined with the Bijvoet method, neither heavy-atom incorporation nor crystallization is necessary for absolute structure determination because heavy atoms (zinc and iodine atoms) are already installed in the sponge framework. In our original report, this great advantage was examined for only one chiral molecule (santonin), which had known configurations at stereogenic carbons. Here the method is applied to the absolute structure analysis of compounds with axial and planar chirality. In addition to standard *o*-substituted biaryl **2** (Fig. 1),‡
‡Crystallographic data for **1**·(*S*)-**2**: C_72_H_48_N_24_Zn_6_I_12_·0.94(C_16_H_16_O_2_), *M* = 3390.77, pale yellow, block, 0.12 × 0.06 × 0.04 mm^3^, monoclinic, space group *C*2, *a* = 34.627(4) Å, *b* = 15.0818(16) Å, *c* = 31.194(3) Å, *β* = 102.7920(10)°, *V* = 15 886(3) Å^3^, *Z* = 4, *D*_c_ = 1.418 g cm^–3^, *T* = 90(2) K, 1.206 < *θ* < 24.760°, *R*_int_ = 0.0408, 1441 parameters, 263 restraints, GoF = 1.045, final *R* factors *R*_1_ = 0.0745, and w*R*_2_ = 0.2548 for all data, Flack parameter = 0.082(11). CCDC deposit number 1051800.
§
§Crystallographic data for **1**·(*R*)-**2**: C_72_H_48_N_24_Zn_6_I_12_·0.85(C_16_H_16_O_2_), *M* = 3370.32, pale yellow, block, 0.12 × 0.08 × 0.08 mm^3^, monoclinic, space group *C*2, *a* = 34.200(3) Å, *b* = 15.1265(13) Å, *c* = 31.003(3) Å, *β* = 102.3850(10)°, *V* = 15 665(2) Å^3^, *Z* = 4, *D*_c_ = 1.429 g cm^–3^, *T* = 90(2) K, 1.219 < *θ* < 26.437°, *R*_int_ = 0.0315, 1307 parameters, 205 restraints, GoF = 1.049, final *R* factors *R*_1_ = 0.0978, and w*R*_2_ = 0.3129 for all data, Flack parameter = 0.169(8). CCDC deposit number 1051799.
7 two chiral molecules obtained in recent asymmetric synthesis studies are analyzed: Yamaguchi and Itami's axially chiral compound **3** (ref. 8) and Mori and Ogasawara's planar chiral compound **4** (Scheme 1).^9^ The successful absolute structure determination, particularly for configurationally unknown **4**, is a fine demonstration that the crystalline sponge method will be of great help for asymmetric synthesis studies when the absolute structures of chiral products are hard to determine.

## Results and discussion

A tiny and high quality single crystal of crystalline sponge [(ZnI_2_)_3_(tpt)_2_·*x*(cyclohexane)]_*n*_ [**1**, tpt = 2,4,6-tris(4-pyridyl)triazine]^10^ was treated with a cyclohexane/1,2-dichloroethane (v/v = 9 : 1) solution of biphenyl compound (*S*)-**2** (5 μg/50 μL).^5^ After incubation at 45 °C for 2 days, the resulting crystal **1**·(*S*)-**2** was subjected to X-ray diffraction analysis. Owing to efficient host–guest interactions with guest (*S*)-**2**, chirality was induced into the originally achiral host framework. The centrosymmetric *C*2/*c* space group of original host **1** changed to non-centrosymmetric *C*2 after guest inclusion. The structure of (*S*)-**2** trapped in the pores was successfully solved in the space group *C*2 and its absolute configuration was confirmed with a Flack parameter of 0.195(13), which was significantly improved to be 0.082(11) when the crystalline sponge was soaked in the guest solution for one month. The data quality improvement was presumably due to the equilibration of the guest binding site in the pore (Fig. 1). When enantiomeric (*R*)-**2** was examined under the same conditions, the mirror image structure was observed with a reasonable Flack parameter [0.169(8)]. The guest occupancies of roughly 50% for **2** were shown to be sufficient for the discrimination of the enantiomers.

**Fig. 2:**
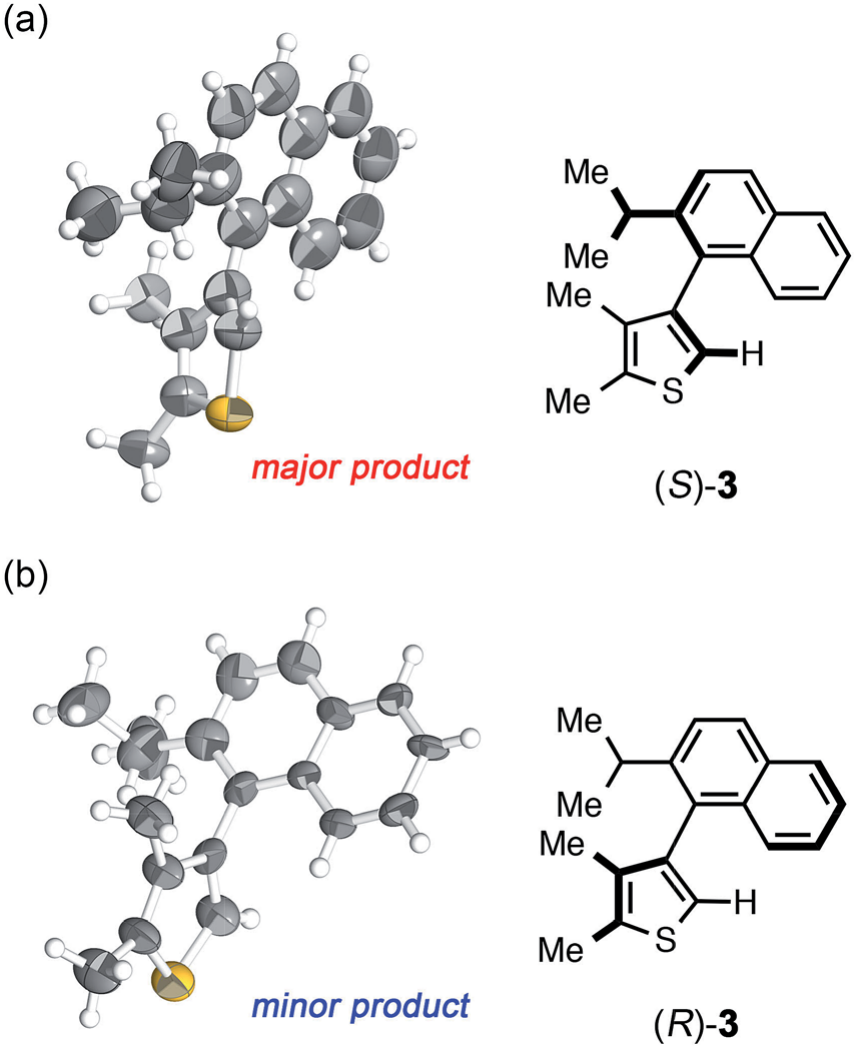
Crystal structure of (a) (*S*)-**3** and (b) (*R*)-**3** determined by the crystalline sponge method (ORTEP at 50% probability).

The crystalline sponge method was next applied to biaryl compound **3**, which was recently prepared by an enantioselective aryl–aryl coupling reaction *via* direct aryl C–H activation (Scheme 1a).^8^ As there are no reliable standard samples for the thiophene–naphthalene biaryl ring systems, it was not easy to determine the absolute configuration of biaryl **3**. As the sponge method can be performed on a microgram scale, racemic **3** was subjected to chiral HPLC separation to obtain enantiomerically pure samples of (*R*)- and (*S*)-**3** in microgram quantities (LC-SCD method). The first fraction (∼5 μg), corresponding to the major enantiomer in the reaction shown in Scheme 1a, was treated with crystalline sponge **1** for 2 days at 50 °C, and the guest-included sponge crystal was subjected to a diffraction study. The crystallographic analysis revealed the absolute structure of (*S*)-**3** with a Flack parameter of 0.102(7) in the monoclinic *C*2 space group. Although the crystal structure contains three crystallographically independent guest molecules, all of them are shown to be in the *S* configuration. The crystalline sponge analysis of the second fraction showed the mirror image of the guest-soaked complex; it was in the *R* configuration with a Flack parameter of 0.046(6), in line with the X-ray crystallographic analysis of a derivative of (*R*)-**3** (Fig. 2).¶
¶Crystallographic data for **1**·(*S*)-**3**: C_72_H_48_N_24_Zn_6_I_12_·0.62(C_19_H_20_S)·0.63(C_6_H_12_), *M* = 3392.41, yellow, block, 0.11 × 0.07 × 0.05 mm^3^, monoclinic, space group *C*2, *a* = 34.4391(11) Å, *b* = 15.0959(3) Å, *c* = 29.9566(10) Å, *β* = 101.454(3)°, *V* = 15 263.4(8) Å^3^, *Z* = 4, *D*_c_ = 1.476 g cm^–3^, *T* = 100(2) K, 3.431 < *θ* < 74.380°, *R*_int_ = 0.0301, 1542 parameters, 438 restraints, GoF = 1.033, final *R* factors *R*_1_ = 0.0749, and w*R*_2_ = 0.2612 for all data, Flack parameter = 0.102(7). CCDC deposit number 1051619.
‖
‖Crystallographic data for **1**·(*R*)-**3**: C_72_H_48_N_24_Zn_6_I_12_·0.5(C_19_H_20_S)·0.5(C_6_H_12_), *M* = 3351.45, yellow, block, 0.13 × 0.11 × 0.09 mm^3^, monoclinic, space group *C*2, *a* = 34.8299(7) Å, *b* = 14.9133(2) Å, *c* = 31.5387(6) Å, *β* = 102.403(2)°, *V* = 15 999.8(5) Å^3^, *Z* = 4, *D*_c_ = 1.391 g cm^–3^, *T* = 100(2) K, 3.236 < *θ* < 74.481°, *R*_int_ = 0.0259, 1521 parameters, 434 restraints, GoF = 1.083, final *R* factors *R*_1_ = 0.0598, and w*R*_2_ = 0.2036 for all data, Flack parameter = 0.046(6). CCDC deposit number 1051618.
8

**Fig. 3:**
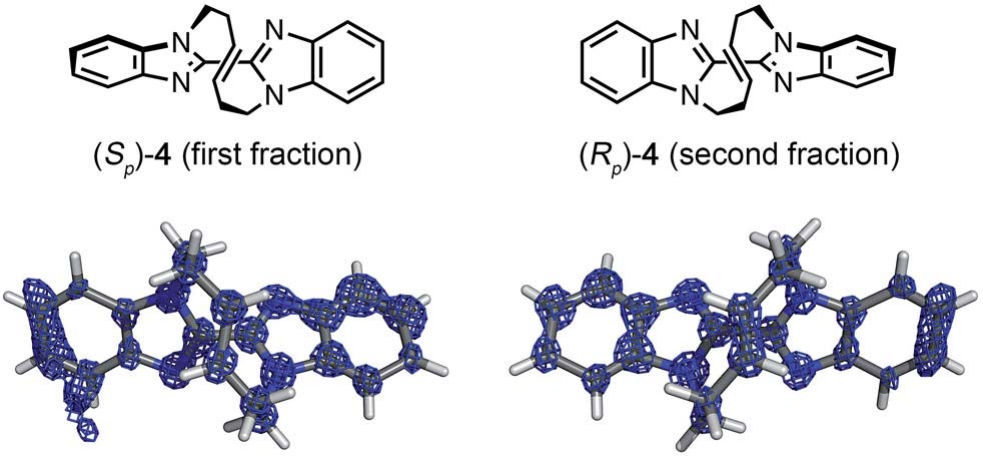
Crystal structures of (*S*_p_)-**4** (first fraction) and (*R*_p_)-**4** (second fraction) superimposed with the electron density map (Fo, counter: 0.6*σ*).

Macrocyclic bisbenzimidazole **4** shows planar chirality because it contains a chiral cyclic *E*-alkene linkage that hardly flips into its enantiomeric structure at room temperature.9*a* The enantioselective synthesis of **4** using a chiral metathesis catalyst has quite recently been developed by Mori and Ogasawara.9*b* Chemical introduction of a heavy atom or a chiral reference group into **4** for absolute structure determination is difficult and, thus, its absolute configuration remained unknown. Again, the crystalline sponge analysis was performed after chiral HPLC separation9*a* of racemic **4**. The first fraction, which corresponded to the major enantiomer in the reaction of Scheme 1b, was separated and subjected to the crystalline sponge method. The crystallographic analysis of the guest-absorbed sponge clearly revealed the *S*_p_ configuration for the major enantiomer of **4** trapped in the pore [Flack parameter: 0.070(5)]. The crystal structure also revealed that the planar chirality of the cyclic *E*-alkene was effectively transferred to the *S* axial chirality of the bisimidazole framework and the *P* helical chirality of the cyclic *E*-alkene framework. Diastereomeric isomers of this compound do not exist because of steric restriction. In a similar fashion, the second (minor) HPLC fraction was determined to be (*R*_p_)-**4** [Flack parameter: 0.044(5)] (Fig. 3).**
**Crystallographic data for **1**·(*S*_p_)-**4**: C_36_H_24_N_12_Zn_3_I_6_·0.5(C_20_H_14_N_4_)·2.4(C_6_H_12_), *M* = 1939.42, colorless, block, 0.27 × 0.1 × 0.08 mm^3^, monoclinic, space group *C*2, *a* = 35.2721(9) Å, *b* = 14.6427(2) Å, *c* = 31.6037(9) Å, *β* = 102.029(2)°, *V* = 15 964.2(7) Å^3^, *Z* = 8, *D*_c_ = 1.614 g cm^–3^, *T* = 100(2) K, 2.562 < *θ* < 77.630°, *R*_int_ = 0.0675, 2127 parameters, 1188 restraints, GoF = 1.015, final *R* factors *R*_1_ = 0.0651, and w*R*_2_ = 0.2139 for all data, Flack parameter = 0.070(5). CCDC deposit number 1043949.
†
†Crystallographic data for **1**·(*R*_p_)-**4**: C_36_H_24_N_12_Zn_3_I_6_·0.27(C_20_H_14_N_4_)·4.4(C_6_H_12_), *M* = 1972.67, colorless, block, 0.22 × 0.07 × 0.07 mm^3^, monoclinic, space group *C*2, *a* = 35.1081(9) Å, *b* = 14.6516(2) Å, *c* = 31.3279(8) Å, *β* = 101.649(2)°, *V* = 15 782.8(6) Å^3^, *Z* = 8, *D*_c_ = 1.660 g cm^–3^, *T* = 100(2) K, 2.570 < *θ* < 77.721°, *R*_int_ = 0.0674, 2285 parameters, 1450 restraints, GoF = 1.021, final *R* factors *R*_1_ = 0.0580, and w*R*_2_ = 0.1852 for all data, Flack parameter = 0.044(5). CCDC deposit number 1043948.


## Conclusions

In conclusion, the crystalline sponge method was successfully applied to the absolute structure determination of compounds with axial and planar chirality. Though the Bijvoet method is one of the most reliable methods for determining absolute configuration, this method is only available for crystalline compounds containing heavy atom(s). Because of this limitation, synthetic chemists tend to avoid the Bijvoet method and to trust empirical methods, such as the Mosher method,^11^ or chemical derivation from or to known chiral compounds. These chemical methods are, however, not effective for chiral compounds without stereogenic centers, such as molecules with axial, planar, or helical chirality. If the two enantiomers of such compounds are separable by analytical chiral HPLC, the sponge method promises the facile determination of the absolute configuration with only microgram quantities of racemic samples. We expect that the sponge method will be widely used, not only in asymmetric synthesis studies but also in various chiral technology fields.

## Guidelines

Special care should be taken for absolute structure determination because of the limitations that are characteristic of the crystalline sponge method. We recommend that users apply the method on the following understanding and guidelines. (1) To observe clear anomalous X-ray scattering from the zinc and iodine atoms of the host, the chiral environment around the zinc and iodine atoms should be created by effective host–guest interactions that induce chiral distortion of the host framework. (2) High guest occupancy is desired for inducing the chiral distortion of the host framework. (3) With weak host–guest interactions and low guest occupancy, pseudo-symmetry problems may arise and it will not be possible to solve the structure in a chiral space group. The method is thus suitable for aromatic or relatively rigid cyclic compounds that can induce both strong host–guest interactions and chiral host distortion. (4) Samples should be enantiomerically pure. When using a 90% ee sample (*i.e.*, 90% pure enantiomer plus 10% racemate), the achiral host, which has racemic binding sites (*C*2/*c*), may preferentially absorb the racemate into the crystal, not providing reliable data. (5) We recommend to analyze both enantiomers, if available, to ensure the absolute structure determination. (6) Because of the low occupancy and the inevitable disorder of the included guest, diffraction at a high angle region is in general weakly observed. Therefore, long exposure time with strong X-ray source is recommended to effectively collect data at a high angle region.

## Experimental

### A typical procedure for guest inclusion into the crystalline sponge

To a microvial containing a single crystal of **1** and cyclohexane (45 μL), 5 μL of a 1,2-dichloroethane solution of the target compound (1 mg/1 mL) was added. The crystal-containing microvial was then allowed to stand at 45 °C and the solvent was gradually evaporated over 2 days according to the previously reported procedure.^5^ The guest inclusion conditions highly depend on the nature of the guest compounds. The detailed procedures for individual compounds are described in ESI.†

### X-ray crystallographic analysis

Obtained guest-included crystalline sponges were subjected to single crystal X-ray diffraction. All the data were collected with an in-house diffractometer using CuKα (*λ* = 1.54184 Å) or MoKα (*λ* = 0.71073 Å) X-ray radiation. All crystal structures were solved using SHELXT^12^ and refined using SHELXL^13^ programs. Details are described in ESI.†

## Supplementary Material

Supplementary information

Crystal structure data
