# Supplementary material for: Absolute structure determination of compounds with axial and planar chirality using the crystalline sponge method
Source: Chem Sci. 2015 May 12;6(7):3765–8. doi: 10.1039/c5sc01681a (PMC5496191; doi:10.1039/c5sc01681a)
Supplement: Supplementary file 1 [file SC-006-C5SC01681A-s001.pdf]

Electronic Supplementary Material (ESI) for Chemical Science.
This journal is © The Royal Society of Chemistry 2015

Supporting Information

Table of Contents

**I.**	**Materials and Methods**	S2–S3
**II.**	**Inclusion of Guest Compounds into Crystalline Sponge**	S4–S12
**III.**	**References**	S13

I. Material and Methods

Reagents and equipment

Solvents and reagents were purchased from TCI Co., Ltd., WAKO Pure Chemical Industries Ltd., and Sigma-Aldrich Co and used without further purification. Crystalline sponge $[(ZnI_2)_3(tpt)_2]_n$ (**1**) was prepared according to the reported procedure.[1] Enantiomerically pure samples of (*R*)- and (*S*)-5,5',6,6'-tetramethyl-1,1'-biphenyl-2,2'-diol (**2**) were prepared from (*R*)- and (*S*)-3,3'-di-*tert*-butyl-5,5',6,6'-tetramethyl-1,1'-biphenyl-2,2'-diol (**2'**), respectively, whose optical purity were confirmed as their (–)-menthyl phosphate forms by ^{31}P NMR to be (>99% *de*).[2,3]

Optically resolved samples of 4-(2-isopropylnaphthalen-1-yl)-2,3-dimethylthiophene (**3**)[4] and di(benzo[*d*]imidazo)[1,2-a:2,1-c]1,8-diaza-7-(E)-cyclodecene (**4**)[5] were provided by the courtesy of Profs. Itami and Yamaguchi (Nagoya University), and Profs. Mori (Kobe University) and Ogasawara (Hokkaido University), respectively. All NMR spectra data were recorded at 300 K and chemical shift values are reported in parts per million (ppm) relative to an internal standard 85% phosphoric acid. Microscopic FT-IR spectra were recorded on a Varian DIGILAB Scimitar instrument and are reported in frequency of absorption (cm^{-1}). Single crystal X-ray diffraction data were collected on Agilent Technologies SuperNova equipped with a cold nitrogen stream low temperature system or a Bruker APEX-II CCD diffractometer equipped with a focusing mirror. For single crystal X-ray diffraction analysis and microscopic IR measurement, fluorolube® and mineral oil were used as a protectant for the single crystals.

X-ray crystallographic analysis

Single crystal X-ray diffraction data collected on a Bruker APEX-II CCD diffractometer equipped with a focusing mirror (MoK_α radiation λ = 0.71073 Å) were processed with Bruker APEX2 software.[6] Absorption correction was performed by empirical method in SADABS.[6] Space groups were determined by XPREP program.[6] Diffraction data measured on a Agilent SuperNova equipped with a CuK_α radiation source (λ = 1.54184 Å) were integrated and scaled using the program CrysAlisPro Version 1.171.37.35.[7] Numerical and empirical absorption corrections are performed during the above process. All structures were solved using a dual-space algorithm (SHELXT[8]) and refined using full-matrix least-squares method (SHELXL[9]). All the non-hydrogen atoms for host framework were refined anisotropically. All hydrogen atoms and some solvents and weakly observed guest molecules were refined isotropically. For refinement details of each inclusion complex, see individual sections.

II. Inclusion of guest compounds into crystalline sponge

General procedure for guest inclusion

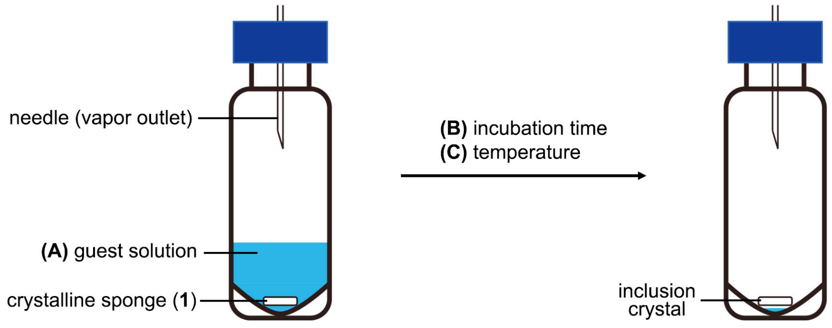

Figure S1. Experimental settings for guest inclusion.

Guest inclusion was performed in a microvial by soaking a crystal of crystalline sponge **1** in a guest solution.[1] The inclusion conditions were optimized by changing (A) the initial guest solution (*i.e.* guest concentration and solvent), (B) incubation time and temperature. The detailed conditions are reported below.

Inclusion complex 1•(*S*)-2

Inclusion conditions:

(A) Compound (*S*)-**2** (5 μg) in cyclohexane/1,2-dichloroethane (v/v = 9:1) 10 μL

(B) The crystal was incubated for 2 days at 45 ºC in the sealed vial, and then the solvent was completely evaporated over 1 day at 4 ºC

Inclusion complex 1•(*S*)-2 (another run)

Inclusion conditions:

(A) Compound (*S*)-**2** (5 μg) in cyclohexane/1,2-dichloroethane (v/v = 9:1) 10 μL

(B) The crystal was incubated for 37 days at 45 ºC in the sealed vial, and then the solvent was completely evaporated over 1 day at 4 ºC

IR (single crystal, mineral oil): 3528, 3053, 2924, 2850, 1616, 1578, 1514, 1468, 1422, 1377, 1316 cm^{-1}.

• Structural analysis detail

Due to the low occupancy of the guest and its disorder with solvent (cyclohexane), anisotropic refinements of guest (*S*)-**2** without any restraints/constraints were unsuccessful. Therefore, based on the known structure of **2**, benzene rings were constrained with AFIX 66 command, and C_{arom}–CH_3 and C_{arom} –OH bond lengths were restrained using SADI with a standard deviation of 0.02. Anisotropic refinement was then performed using SIMU with a standard deviation of 0.02 for guest molecules (*S*)-**2**. Solvents could not be reasonably modeled because of severe disorder.

Inclusion complex 1•(*R*)-2

Inclusion conditions:

(A) Compound (*R*)-**2** (5 μg) in cyclohexane/dichloromethane (v/v = 9:1) 50 μL

(B) The solvent was completely evaporated over 2 days at 45 ºC

IR (single crystal, mineral oil): 3495, 3053, 2918, 2851, 1616, 1576, 1529, 1466, 1423, 1375, 1316 cm^{-1}.

• Structural analysis detail

For similar reasons, the geometries of the partial structures of the guest were constrained with AFIX 66 for the aromatic rings, and restrained with SADI, FLAT, and DFIX commands with standard deviations of 0.02, 0.1, and 0.02, respectively based on the known structure. Anisotropic refinements of (*R*)-**2** were performed using SIMU and DELU with a standard deviation of 0.02 and partly with a constraint EADP.

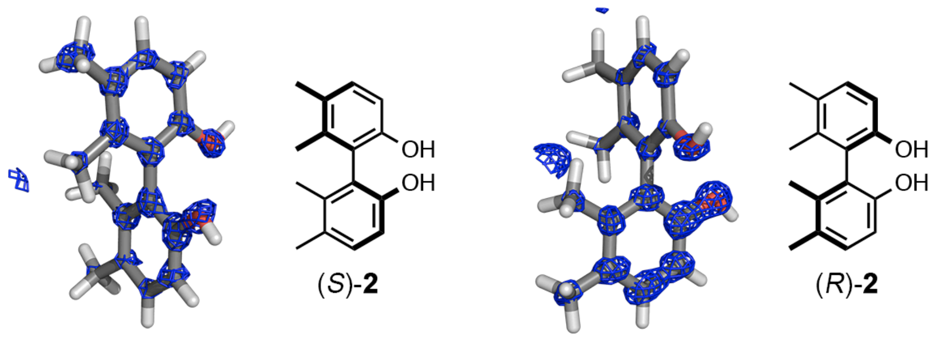

Figure S2. The observed electron density map (F_o, contour: 0.6σ) around the included (*S*)-**2** (left) and (*R*)-**2** (right).

Inclusion complex 1•(*S*)-3

Inclusion conditions:

(A) Compound (*S*)-**3** (5 μg) in cyclohexane/dichloromethane (v/v = 9:1) 20 μL

(B) The solvent was completely evaporated over 12 h, and then the crystal was allowed to stand for 36 h in the resultant oil at 50 ºC.

IR (single crystal, mineral oil): 3358, 3054, 2953, 2855, 1672, 1618, 1576, 1523, 1423, 1374, 1316 cm^{-1}.

Inclusion complex 1•(*R*)-3

Inclusion conditions:

(A) Compound (*R*)-**3** (5 μg) in cyclohexane/dichloromethane (v/v = 9:1) 20 μL

(B) The solvent was completely evaporated over 12 h, and then the crystal was allowed to stand for 36 h in the resultant oil at 50 ºC.

IR (single crystal, mineral oil): 3356, 3054, 2930, 2852, 1672, 1618, 1578, 1530, 1423, 1378, 1316 cm^{-1}.

• Structural analysis detail for **1•**(*S*)**-3** and **1•**(*R*)**-3**

The framework, including disorder of ZnI_2 parts, was properly refined without using restraint and constraint. After the refinement of the framework only, Flack parameters obtained by Persons method reached to below 0.15 [χ = 0.120(7) for **1**•(*S*)-**3**, χ = 0.146(6) for **1**•(*R*)-**3**]. These values were clear evidence about that chirality of the guest compound is represented as a contrast of Bijvoet pairs in the reflection data sets with help from strong anomalous scattering of zinc and iodine atoms in the framework. Models of guest structure for refinement were built by simply assigning proper electron density peaks to non-hydrogen atoms at this stage. Especially, the sulfur atom was easily found because the position of this atom was clearly represented as a relatively strong electron density peak. However, these models were unstable in least-square refinements and easily diverged after some refinement cycles. One reason for the divergence is the low population of the guest compound in the crystal. In both crystal structures, a guest compound was placed on the two-fold symmetry element and its population in an asymmetric unit cannot be beyond 50%. Another reason would be unassignable residual electron densities in a void space. Many electron density peaks

with amplitude of ca. 1.0 e Å^{-3} were strewn in this space. This amplitude is almost same as that of electron density peaks assigned as carbon atoms in a guest molecule. Therefore, the guest's carbon positions were not converged at suitable positions in the least-square refinement. For managing this unstable feature in refinement, restraints (DFIX, DANG, SADI, SAME, FLAT, SIMU, ISOR, with reduced standard deviation in some cases) and constraint for making a naphthalene ring were applied in structure analyses. Population of guest of two independent (*S*)-**3** molecules were estimated as 37.1(6)% and 25.1(8)% from the refinement of the guest/solvent disorder model. In this refinement, cyclohexane molecules were refined using some restraints (DFIX, DANG, SIMU, ISOR) with default value of their standard deviations. Regarding the two independent (*R*)-**3** molecules in the crystal, their populations were estimated as 27.8(4)% and 22.2(4)% from the refinement of disorder model of overlapped two guest molecules.

We tried to make a suitable model describing in this void space for removing the above restrictions toward a guest structure in structural refinement. In the void space, an electron density peak corresponding to the sulfur atom of the guest, which is expected to appear with significant amplitude (higher than 1.0 e Å^{-3}, typically 3-4 e Å^{-3}), was not observed. From this observation, we considered that this void space was filled by solvent molecules only. However, due to the messy electron density in this space, we could not make a suitable model for the refinement of disordered solvent molecules. Therefore, we could not remove the above restrictions for the structural analysis. Even under these restricted conditions, Flack parameters obtained Persons method were reached to 0.102(7) [**1**•(*S*)-**3**] and 0.046(6) [**1**•(*R*)-**3**]. We consider that these data and crystal structures show correct absolute configuration of the target compounds.

Inclusion complex 1•(S_p)-4

Inclusion conditions:

(A) Compound (S_p)-**4** (5 μg) in cyclohexane/dichloromethane (v/v = 9:1) 80 μL

(B) The solvent was completely evaporated over 2 d at 50 ºC

IR (single crystal, mineral oil): 3053, 2924, 2847, 1618, 1577, 1526, 1450, 1421, 1373, 1317 cm^{-1}.

Inclusion complex 1•(R_p)-4

Inclusion conditions:

(A) Compound (***R**$_p$*)-**4** (5 μg) in cyclohexane/dichloromethane (v/v = 9:1) 80 μL

(B) The solvent was completely evaporated over 2 d at 50 ºC

IR (single crystal, mineral oil): 3054, 2950, 2852, 1618, 1577, 1524, 1451, 1420 1377 1317 cm^{-1}.

• Structural analysis detail for **1**•(S_p)-**4** and **1**•(R_p)-**4**

Crystal structures of **1**•(S_p)-**4** and **1**•(R_p)-**4** were successfully solved as guest/solvent disordered models. The tpt ligands in the framework were refined without restraints and constraints. The ZnI_2 parts were refined as a disorder model using some restraints (SIMU, SADI, SAME) with default value of their standard deviations. In the guest/solvent disorder models of both crystals, some solvated cyclohexane molecules were overlapped with the guest fragment. This overlap led to geometrical distortion and non-positive definite of atomic displacement parameters in the refinement of guest fragment. Therefore, minimum numbers of restraint (SIMU, DFIX) were applied using default value of their standard deviations. Some benzene ring parts in both compounds were fixed as the idealized geometry using the AFIX 66 command. Populations of guest molecules in both crystals were estimated by the refinement of guest/solvent disorder models as 49.1(4)%, 50.9(4)% [two independent (S_p)-**4** molecules], and 53.1(7)% [(R_p)-**4**]. In this disorder models, cyclohexane molecules were refined using some restraints (DFIX, DANG, SIMU, ISOR) with default value of their standard deviations.

References

[1] Y. Inokuma, S. Yoshioka, J. Ariyoshi, T. Arai, M. Fujita, *Nat. Protoc.* **2014**, *9*, 246-252.

[2] Z. Hua, V. C. Vssar, I. Ojima, *Org. Lett.* **2003**, *5*, 3831-3834.

[3] J. B. Alexander, R. R. Shrock, W. M. Davis, K. C. Hultzsch, A. H. Hoveyda, J. H. Houser *Organometallics* **2000**, *19*, 3700-3715.

[4] K. Yamaguchi, J. Yamaguchi, A. Studer, K. Itami, *Chem. Sci.* **2012**, *3*, 2165-2169.

[5] Y. Okayama, S. Tsuji, Y. Toyomori, A. Mori, S. Arae, W. Wu, T. Takahashi, M. Ogasawara, *in press* (DOI: 10.1002/anie.201500459).

[6] Bruker (2007), *APEX2*, *SADABS* and *XPREP*, Bruker AXS Inc., Madison, Wisconsin, USA.

[7] Agilent (2014), CrysAlisPro Version 1.171.37.35, Agilent Technologies UK Ltd, Yarnton, England.

[8] G. M. Sheldrick, *Acta Crystallogr. Sect. A* **2015**, 71, 3-8.

[9] G. M. Sheldrick, *Acta Crystallogr. Sect. C* **2015**, 71, 3-8.
